# SFE-DETR: An Enhanced Transformer-Based Face Detector for Small Target Faces in Open Complex Scenes

**DOI:** 10.3390/s26010125

**Published:** 2025-12-24

**Authors:** Chenhao Yang, Yueming Jiang, Chunyan Song

**Affiliations:** School of Computer and Control Engineering, Northeast Forestry University, Harbin 150040, China; ych428@nefu.edu.cn (C.Y.); songchunyan@nefu.edu.cn (C.S.)

**Keywords:** object detection, small face detection, feature extraction, feature fusion pyramid, RT-DETR, open complex scenes

## Abstract

Face detection is an important task in the field of computer vision and is widely applied in various applications. However, in open and complex scenes with dense faces, occlusions, and image degradation, small face detection still faces significant challenges due to the extremely small target scale, difficult localization, and severe background interference. To address these issues, this paper proposes a small face detector for open complex scenes, SFE-DETR, which aims to simultaneously improve detection accuracy and computational efficiency. The backbone network of the model adopts an inverted residual shift convolution and dilated reparameterization structure, which enhances shallow features and enables deep feature self-adaptation, thereby better preserving small-scale information and reducing the number of parameters. Additionally, a multi-head multi-scale self-attention mechanism is introduced to fuse multi-scale convolutional features with channel-wise weighting, capturing fine-grained facial features while suppressing background noise. Moreover, a redesigned SFE-FPN introduces high-resolution layers and incorporates a novel feature fusion module consisting of local, large-scale, and global branches, efficiently aggregating multi-level features and significantly improving small face detection performance. Experimental results on two challenging small face detection datasets show that SFE-DETR reduces parameters by 28.1% compared to the original RT-DETR-R18 model, achieving a mAP50 of 94.7% and AP-s of 42.1% on the SCUT-HEAD dataset, and a mAP50 of 86.3% on the WIDER FACE (Hard) subset. These results demonstrate that SFE-DETR achieves optimal detection performance among models of the same scale while maintaining efficiency.

## 1. Introduction

Face detection serves as the core and foundational stage for a wide range of face-related applications, including face recognition [1], identity authentication, and facial attribute analysis [2], providing a critical prerequisite for the implementation and advancement of subsequent applications. As an essential basis for personal identity identification, facial information exhibits characteristics of relative stability and uniqueness. However, in densely populated and complex environments—such as transportation hubs or crowded roads during holidays—faces captured by surveillance devices are often degraded by occlusion, illumination variations, and reflective interference. These factors significantly reduce facial clarity, making pedestrian facial features difficult to discern and thereby posing challenges for subsequent identity verification and event investigation. Such difficulties can hinder practical tasks including criminal investigation, prevention of stampede incidents, and identification of potential fire safety hazards.

Images acquired in open and complex scenarios by devices such as surveillance cameras and UAVs exhibit substantial diversity. In such scenarios, target individuals do not cooperate with the imaging devices during data acquisition, resulting in environments with minimal constraints [3]. Consequently, the captured images present wide variations in illumination conditions, viewing angles, and background complexity, and are heavily affected by image degradation factors such as noise, camera shake, and motion blur. Moreover, to achieve a wide field of view, these acquisition devices are typically positioned at a considerable distance from the target crowd, leading to small-scale face instances in the captured images. Small face images are particularly vulnerable to degradation caused by noise, jitter, and blur, which further exacerbates the difficulty of reliable detection. As a result, existing methods in the domain of small-face detection continue to face significant technical challenges and performance bottlenecks. As a representative application of generic object detection, face detection techniques have benefited substantially from recent advances in object detection frameworks. In particular, two-stage models such as Faster R-CNN [4] and Mask R-CNN [5], as well as one-stage models including SSD [6], RetinaNet [7], and the YOLO-series models [8], have provided a solid foundation for the development of various face detection approaches.

Two-stage detection algorithms generate candidate regions through a region proposal network and subsequently select the best-matching regions as targets. Although such methods generally achieve high detection accuracy, their inference speed is relatively slow. In contrast, one-stage detectors employ end-to-end convolutional neural networks for object detection, maintaining competitive accuracy while offering advantages in terms of high speed and real-time performance. However, these methods typically rely on non-maximum suppression (NMS) as a post-processing step. In densely populated target scenarios, the computational burden of NMS increases significantly, leading to reduced detection efficiency. Furthermore, challenges such as small object sizes, multi-scale overlap, and complex background interference make it difficult for conventional convolutional kernels to effectively capture fine-grained features and spatial relationships, thereby degrading detection accuracy. Transformer-based models [9] exhibit inherent advantages in addressing these issues. DETR [10] Innovatively integrates convolutional neural networks with Transformer architectures, enabling end-to-end learning to directly predict object locations and categories. By eliminating anchor-based localization and NMS procedures commonly used in traditional approaches, DETR-based models improve operational efficiency while maintaining detection accuracy. Nevertheless, DETR and related models still suffer from high computational costs, prolonged training time, and relatively slow inference speed.

The introduction of RT-DETR [11] has effectively addressed this bottleneck by significantly improving inference speed while maintaining high detection accuracy. Its core innovation lies in the design of an efficient hybrid encoder, which decouples same-scale interactions from cross-scale fusion mechanisms to enable efficient multi-scale feature processing. Nevertheless, as a general-purpose object detection framework, DETR-based models have not demonstrated pronounced advantages in small-object detection. This limitation has motivated a series of targeted improvements. To enhance small-object detection performance, Google proposed ViDT [12], the first pure Transformer-based detector, which replaces the conventional ResNet backbone with a Vision Transformer, resulting in substantial improvements in feature extraction capability and small-scale object recognition. SOF-DETR [13], proposed by the team led by Shikha Dubey, further strengthens small-object detection by fusing third- and fourth-stage convolutional features into the decoder input. AMFEF-DETR [14] introduces a frequency-adaptive mechanism into the backbone network, effectively enhancing the representation of small objects. ACD-DETR [15] designs an adaptive cross-scale architecture to improve boundary semantic fusion, specifically targeting small-object detection in UAV scenarios, and achieves notable progress in model lightweighting and deployment feasibility. However, existing methods generally face a trade-off between performance and efficiency. High-performance models often incur prohibitive deployment costs due to their computational complexity, whereas lightweight solutions tend to suffer from insufficient detection capability in complex real-world scenarios.

Therefore, this study adopts RT-DETR as the baseline model and proposes a novel small-face detection method, termed SFE-DETR, built upon this framework. Motivated by the observation that small-object features are predominantly concentrated in shallow feature maps [16] and that standard convolutions suffer from redundant computations [17], we design a new backbone network named ISD-Net. In ISD-Net, the extraction accuracy of small-object features is enhanced at shallow layers by strengthening the interaction between local and global information, while lightweight characteristics and stable feature representations are preserved at deeper layers. Furthermore, by incorporating the proposed MHMSA mechanism, a dual-branch architecture is constructed to integrate channel attention with multi-scale feature extraction strategies, effectively improving high-level feature representation capability. In addition, the proposed method introduces an SFE-FPN structure that integrates a carefully designed CSPO-Fusion module. The core components of this module employ three complementary branches—Local, Large, and Global—to achieve more efficient feature map fusion, thereby significantly improving the detection accuracy of small face targets.

The main contributions of this work are summarized as follows:

1. We propose a new backbone network, ISD-Net, which enhances local–global feature interaction in the shallow layers and optimizes multi-scale facial feature representation in the deeper layers. This design effectively reduces redundant computation while improving fine-grained feature characterization, thereby maintaining a lightweight structure without sacrificing representational capacity;
2. A multi-head multi-scale self-attention (MHMSA) mechanism is innovatively introduced, which integrates multi-scale feature modeling with channel attention strategies. This design significantly enhances the model’s ability to extract fine-grained details of multi-scale targets in complex scenarios, while effectively suppressing background interference.
3. We propose a novel feature fusion pyramid network, termed SFE-FPN, whose core design focuses on capturing fine-grained details of small face targets and enhancing their feature representations. By incorporating previously underutilized hierarchical information together with innovatively designed large-kernel convolutions and a dual-domain attention mechanism, the proposed SFE-FPN effectively expands the receptive field while preserving discriminative information of small face targets.

## 2. Related Work

### 2.1. Small Face Detection Methods

Existing small-face detection methods primarily focus on anchor design, multi-scale feature representation, context information fusion, and model lightweighting. However, they still face substantial challenges in real-world scenarios with complex backgrounds. FaceBoxes [18] targets real-time detection by aggressively compressing feature representations to reduce computational overhead. Although this strategy significantly improves detection speed, the limited preservation of fine-grained information degrades its performance in detecting small-scale faces and handling densely crowded scenes. DSFD [19] and the method proposed by Zhu et al. [20] improve detection performance by optimizing anchor distribution and matching strategies, enabling small-scale faces to be more effectively perceived during training. Nevertheless, such approaches typically require generating a large number of candidate regions, which may introduce additional interference in complex backgrounds and consequently increase computational and post-processing burdens. SSH [21] and the low-light face detection method proposed by Wang et al. [22] enhance robustness to occlusion and illumination variations by incorporating richer contextual information. However, when scene structures vary or background information becomes highly complex, the strong reliance on environmental cues may lead to unstable localization or increased false positives. Moreover, their training processes are relatively complex. YOLO5Face [23] improves the localization accuracy of small faces to some extent by introducing facial landmark constraints and modifying the network architecture. However, the multi-task design increases overall model complexity, and its performance gains remain limited when dealing with very small faces or incomplete landmark information. Overall, while existing methods improve small-face detection accuracy, they often require a trade-off between detection performance and runtime efficiency. This limitation becomes particularly pronounced under complex environmental conditions and challenging sample distributions.

### 2.2. Detection Transformer

Carion et al. [10] first proposed DETR, a Transformer-based end-to-end object detector, which has attracted considerable attention due to its distinctive characteristics. DETR optimizes model parameters through end-to-end training, enabling accurate object detection directly from images. Benefiting from the global self-attention mechanism, DETR eliminates the need for post-processing steps such as non-maximum suppression (NMS) during both training and inference. Despite these advantages, DETR suffers from slow training convergence, high computational cost, and difficulties in query optimization. To address these issues, DAB-DETR [24] and DN-DETR [25] introduce iterative refinement schemes and denoising training strategies, respectively, to improve detection performance and accelerate convergence. Group DETR [26] revisits the slow convergence issue of DETR and reveals that the one-to-one label assignment strategy adopted by DETR partially contributes to this limitation. Accordingly, a novel group-based many-to-one label assignment strategy is proposed for the DETR family. Efficient DETR [27] enhances decoder queries by selecting the top-K positions from dense encoder predictions, thereby reducing computational cost. Deformable DETR [28] introduces a deformable attention module that transforms densely connected attention into learnable sparse attention. This design not only enables multi-scale feature detection but also significantly reduces computational complexity. Conditional DETR [29] and Anchor DETR [30] alleviate the difficulty of query optimization, while DINO [31] proposes a hybrid query selection strategy to improve query initialization. Although these variants substantially improve various aspects of DETR-based detectors, their real-time performance still lags behind that of one-stage detectors. In contrast, RT-DETR [11] inherits the strong global modeling capability of Transformers while enhancing detection efficiency through an efficient hybrid encoder. This advantage constitutes the primary motivation for selecting RT-DETR as the baseline model in this study.

## 3. Materials and Methods

### 3.1. Overall Structure

**Figure 1:**
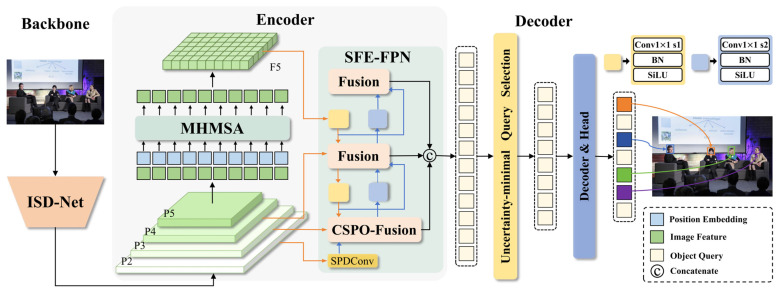
Overall structure of SFE-DETR.

This study proposes a small-face detection method based on SFE-DETR, and the overall architecture is illustrated in Figure 1. The ISD-Net backbone adopts a dual-module reconstruction strategy. Specifically, the shallow layers are designed to effectively enhance the interaction between local and global features, while the deep layers expand the receptive field and perform multi-scale convolutional fusion, thereby significantly reducing parameter redundancy and providing high-quality feature representations for subsequent modules. In the encoder, a multi-head multi-scale self-attention (MHMSA) mechanism is introduced. Through a dual-branch structure that integrates channel attention with multi-scale feature extraction, MHMSA effectively compensates for the degradation in small-object detection accuracy and localization caused by conventional global interaction mechanisms. To address the limitations of the RT-DETR fusion module in handling geometrically deformed targets, we propose SFE-PFN. Specifically, the P2 feature processed by the SPDConv [32] module is fed into the next stage, and a novel feature fusion module, termed CSPO-Fusion, is designed. The CSPO module, built upon large-kernel convolutions and dual-domain attention mechanisms, consists of three complementary branches—Global, Large-scale, and Local—which enable more efficient learning of multi-scale local and global features, thereby improving the detection performance of small face targets.

### 3.2. Inverted Residual Shift-Wise Dilated-Reparam Backbone

**Figure 2:**
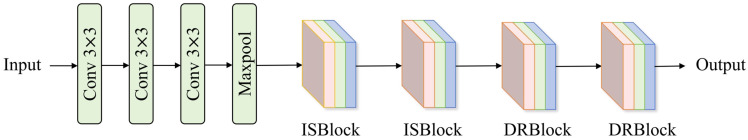
Represents the ISD-Net backbone network.

In open and complex scenarios, sensors such as surveillance cameras and UAVs are constrained by hardware limitations, and small, localized face targets within their fields of view are often missed due to weak feature saliency or low spatial occupancy. Although ResNet-18, as a lightweight backbone network, is suitable for deployment on edge devices, its limited capability in capturing shallow features and insufficient adaptability of deep receptive fields make it difficult to meet the accuracy requirements of face detection in complex environments. To address these limitations, this study proposes a novel backbone network, termed ISD-Net (as illustrated in Figure 2), which consists of two newly designed modules: the IS module and the DR module. The IS module employs local self-attention and multi-branch convolutional structures to effectively preserve fine-grained information of small targets while controlling computational overhead, thereby enhancing the stability of shallow feature representations. The DR module expands the deep receptive field through equivalent large-kernel convolutions, strengthening semantic representation capability while avoiding parameter inflation. By collaboratively operating from fine-grained details to high-level semantics, these two modules achieve continuous feature enhancement, effectively meeting the requirements of small face detection in complex scenarios.

**Figure 3:**
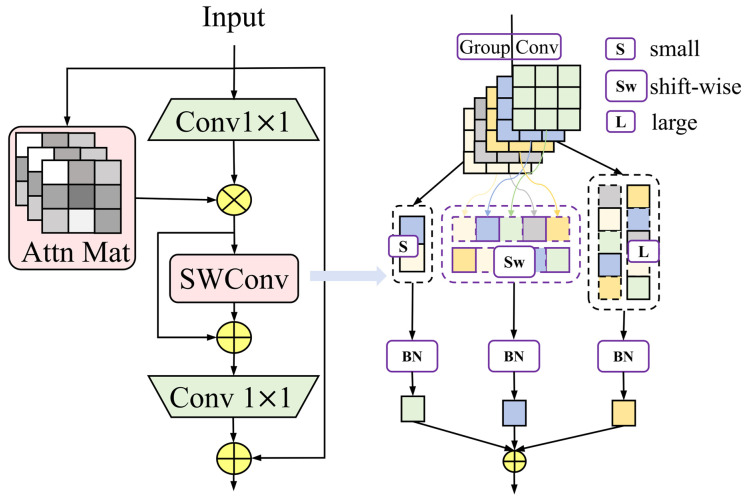
Represents the ISBlock.

Specifically, the IS module (as illustrated in Figure 3) introduces controllable self-attention interactions within local windows through Window-based Multi-Head Self-Attention (Window−MHSA). This design enables shallow features to be jointly processed and fused, thereby capturing both local fine-grained details and global semantic information, as formulated in Equation (1). Here, $X_{attn}$ denotes the output feature map obtained after the attention-based fusion. Subsequently, the SWConv block adopts a parallel design composed of a large-kernel convolution branch, a decomposed-convolution branch, and a small-kernel convolution branch, which further strengthens fine-grained edge details. Specifically, the large-kernel branch enlarges the receptive field, whereas the decomposed-convolution branch simulates a large-kernel convolution via a sequence of sliding small-kernel convolutions, thereby reducing computational overhead. The small-kernel branch supplements local detail features and, together with skip connections, prevents excessive loss of shallow information during processing. In this way, the visibility and discriminability of small targets in early layers are fundamentally improved. The overall process is formulated in Equations (2)–(5).

(1) $$X_{attn}=Window-MHSA(Q,K,V)⊗Conv_{1}(X_{in})$$

(2) $$y_{larae}=Conv_{k\times k}(X_{attn}),k\gg 3$$

(3) $$y_{sw}=shift-wise(Conv_{s\times s}(X_{attn}))$$

(4) $$y_{small}=Conv_{s\times s}(X_{attn}),s<k$$

(5) $$Y_{SW}=SiLU(BN(y_{main}+y_{small})),y_{main}=\left\{\begin{array}{c} y_{sw},\;\;\;\;\;\;Decom=True\\ y_{large},\;\;\;\;Decom=False\;\;\; \end{array}\right.$$

Here, $y_{larae}$ denotes the large-kernel convolution, $y_{sw}$ represents the decomposed convolution, $y_{small}$ indicates the small-kernel convolution, and $Y_{SW}$ refers to the sliding simulation process.

**Figure 4:**
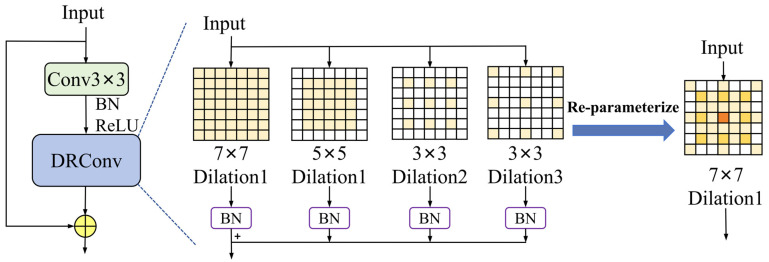
Represents the DRBlock.

Building upon this design, the DR module (as illustrated in Figure 4) is responsible for receptive field adaptation and semantic enhancement in the later stages. By combining non-dilated small kernels with multiple dilated small kernels, it provides an effective replacement for large-kernel convolutions, enabling deep features to obtain a larger and more flexible effective receptive field under controlled parameter complexity. This design allows better coverage of small-face targets and their surrounding contextual information, thereby alleviating semantic representation bias caused by insufficient receptive fields or scale mismatch in deep layers, as formulated in Equation (6).

(6) $$Y_{DR}^{Rep}=BN\left(DConv_{7}(X_{in})\right)+\sum_{\left(r,k\right)}\;BN(X_{in}\cdot \;DConv_{r,k}(X_{in}))$$

Here, $DConv_{r,k}$ denotes convolutional weights with parallel dilation rates r and kernel sizes k, where $r,k\in \left\{\left(5,\;1\right),\left(3,\;2\right),\left(3,\;1\right)\right\}$, which are ultimately transformed into an equivalent large kernel with K = 7. The proposed ISD-Net backbone is designed to preserve shallow features of small targets before they are overwhelmed by deep representations, and to effectively integrate these retained cues with broader contextual information at deeper stages. As a result, the backbone achieves a continuous enhancement from fine-grained shallow details to high-level semantic context, thereby addressing the dual bottlenecks of the original backbone in small-face detection tasks.

### 3.3. The Multi-Head Multi-Scale Self-Attention Mechanism

**Figure 5:**
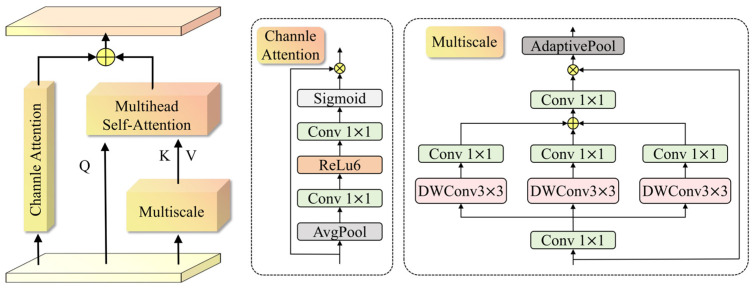
Illustration of the MHMSA mechanism.

Although the MSA structure in the AIFI module is capable of effectively aggregating global information, in open and complex scenarios its attention allocation is often dominated by background regions and large-scale objects. This tendency causes the attention weights to become overly uniform, leading to the attenuation of fine-grained features of small-face targets during deep feature fusion. To address this limitation, we propose the multi-head multi-scale self-attention (MHMSA) mechanism (as illustrated in Figure 5), which preserves and enhances small-face features through a collaborative mechanism that integrates multi-scale contextual modeling with channel-attention enhancement. 

The MHMSA mechanism adopts a dual-branch architecture. The multi-scale information extraction branch employs parallel depthwise separable dilated convolutions to construct receptive fields at different scales. This design introduces global contextual information while simultaneously retaining the local detail features critical for small-face representation, thereby mitigating the feature-smoothing effect caused by single-scale global aggregation. The fused multi-scale features are further processed by a multi-head self-attention mechanism, enabling global interaction and alignment across different scales and enhancing the model’s capability to capture the relationships between small targets and large-scale contextual regions. In parallel, the channel-attention branch dynamically reweights feature channels to emphasize face-related discriminative channels while suppressing background noise channels. From the perspective of feature representation, this mechanism reduces the bias of global attention toward irrelevant information. Acting synergistically along the spatial-scale and channel-semantic dimensions, the two branches enable fine-grained small-face details to be more effectively integrated into deep semantic features. As a result, the proposed MHMSA module alleviates the dilution of small-target features inherent in conventional MSA structures and improves the model’s perceptual robustness in complex scenarios. The processing flow of the MHMSA module can be formulated as follows:

(7) $$Ms=AdaptivePool(Conv_{1}(\sum_{i=1}^{3}\;Conv_{1}(DWConv_{3}(Conv_{1}(X_{in})))))⊗X_{in})$$

(8) $$(Q,K,V)=\left(X_{in}W^{q},MsW^{k},MsW^{v}\right)$$

(9) Attention=MSAttn(Q,K,V)

(10) $$Attention_{c}=Sigmoid(Conv_{1}(ReLu6(Conv_{1}\cdot Avgpool(X_{in}))))⊗X_{in}$$

(11) $$F5=Attention+Attention_{c}$$

where $X_{in}$ stands for P5 layer features, Ms stands for multi-scale convolutional feature extraction, and AdaptivePool stands for adaptive pooling, where $W^{q}$, $W^{k}$ and $W^{v}$ represent the linear transformation weight matrices used to generate query, key and value tensors to accommodate the multi-head self-attention mechanism, respectively. Attention is the feature output of the multi-head self-attention mechanism, while $Attention_{c}$ is the weighted output of the channel attention. As the final feature map, F5 combines the multi-scale fused attention information with channel attention information as the top-level output to provide support for subsequent construction of the feature pyramid.

### 3.4. The SFE-FPN Module

**Figure 6:**
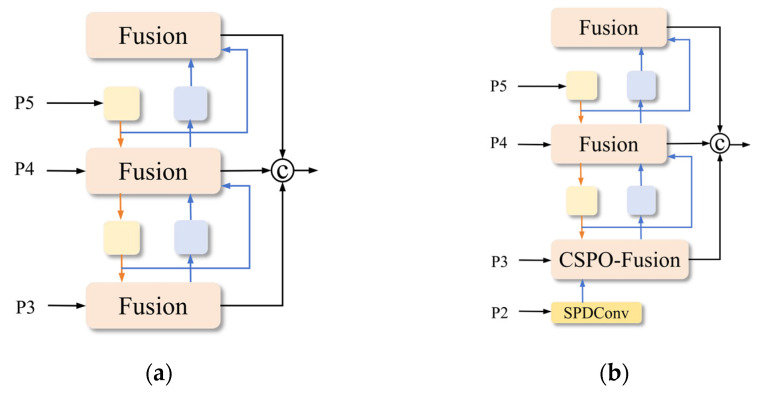
Illustration of the different FPN architecture. (**a**) FPN; (**b**) SFE-FPN.

Traditional models commonly employ a Feature Pyramid Network (FPN) as the hybrid encoder for multi-scale object detection. However, as illustrated in Figure 6a, the conventional FPN only fuses features from the last three pyramid levels (P3, P4, and P5), while neglecting the rich small-target information contained in lower-level feature maps. To better exploit the fine-grained details of small-face targets embedded in shallow features, several studies [33,34] introduce an additional P2 layer, extending the standard three-level FPN architecture [35] to a four-level design. Nevertheless, this modification results in a substantial increase in model parameters and computational complexity. To address this issue, we propose the SFE-FPN architecture, as shown in Figure 6b, which efficiently extracts small-face detail features without significantly increasing the parameter count.

**Figure 7:**
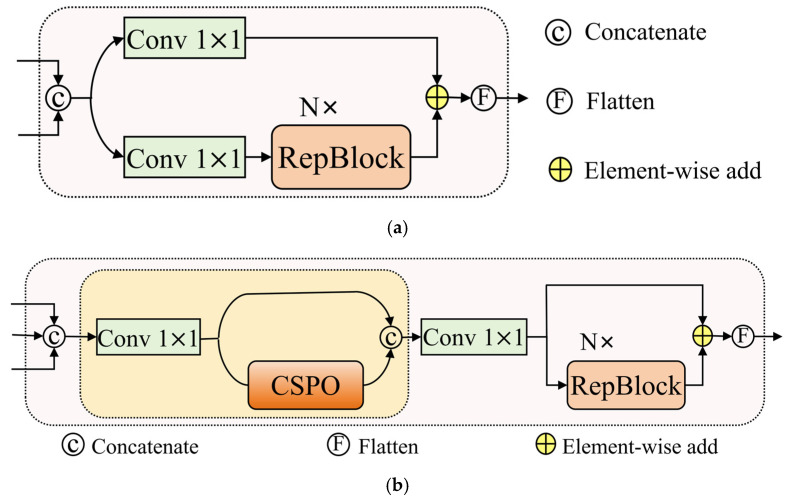
Illustration of the different fusion blocks. (**a**) Fusion; (**b**) CSPO-Fusion.

In traditional convolutional networks, downsampling is typically achieved through strided convolutions or pooling operations. Such approaches directly discard a large amount of spatial information, causing targets with already small scales to be rapidly weakened at shallow stages. In contrast, SPDConv maps the spatial information of the P2 layer into the depth dimension by increasing the channel capacity, thereby preserving critical information. By employing non-strided convolutions to retain fine-grained details and align channel representations, SPDConv effectively mitigates the loss of subtle features and fully preserves the original pixel-level information, providing more complete and stable feature inputs for subsequent deep modeling. In addition, conventional fusion modules (as illustrated in Figure 7a) tend to overemphasize shallow features, which may dominate and obscure deep semantic representations. To fully exploit the rich small-target information contained in lower-level features, we design a novel feature fusion module termed CSPO-Fusion. As illustrated in Figure 7b, this module follows a hierarchical processing strategy. The input features are first processed by a 1 × 1 convolution and then refined through the CSPO module to enhance cross-stage information interaction and feature extraction. After feature concatenation, convolutional operations are applied, followed by deep feature reconstruction using a RepBlock. Finally, the output features are fused via element-wise addition, which preserves the propagation of shallow details while strengthening deep semantic representations. Overall, the proposed design not only avoids the loss of small-target details caused by conventional downsampling but also suppresses the tendency of feature fusion to overly favor shallow information. Consequently, the output features simultaneously exhibit more complete fine-grained structural details and stronger deep semantic discriminability.

**Figure 8:**
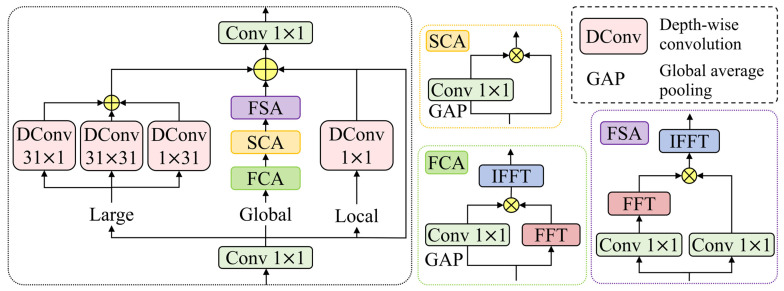
Illustration of the CSPO module.

The core CSPO module in the new feature fusion module is a network feature extraction module. It adopts a combined strategy of multi-scale receptive field modeling and dual-domain attention enhancement, enabling more accurate representation of small-target features, as illustrated in Figure 8. Owing to the limited effective receptive field attainable by conventional convolutions at finite network depth, the contextual relationships between small targets and their surrounding regions are often insufficiently modeled. To address this issue, the CSPO module constructs receptive fields at different scales through three parallel paths—namely Local, Large, and Global—thereby avoiding the scale bias introduced by single-path designs. In recent years, large-kernel convolutions have demonstrated remarkable performance in various image processing tasks, such as semantic segmentation and image restoration. In the Large branch, square large-kernel depth-wise convolutions are employed to efficiently construct an expanded receptive field, enlarging contextual coverage without a significant increase in computational cost. This design alleviates the receptive field limitations caused by finite network depth and suppresses the degradation of low-level details induced by excessive inter-layer abstraction. In parallel with the square-kernel convolution, a strip-shaped depth-wise convolution branch is introduced to capture stripe-like contextual information at a lower computational cost, further enhancing the spatial representation of small targets in complex backgrounds. The complementarity between square large kernels and strip-shaped kernels enables the model to acquire broad contextual information while avoiding excessive smoothing that could compromise fine-grained small-target details.

Background noise often obscures the high-frequency edges and texture responses of small targets, whereas frequency-domain processing is inherently more effective at modeling long-range dependencies and fine-grained details. To address this challenge, the Global branch introduces a dual-domain processing mechanism that integrates the complementary advantages of the spatial and frequency domains. Specifically, a Frequency-domain Channel Attention (FCA) module is employed, in which one path maps feature representations into the frequency domain via the Fast Fourier Transform (FFT) and applies channel-wise weighting, while the other path utilizes global average pooling followed by convolution to generate channel attention weights for the frequency-domain features. The fused representations are then transformed back to the spatial domain through the Inverse Fast Fourier Transform (IFFT), where high-frequency edge and texture components are emphasized and redundant low-frequency background responses are suppressed from a spectral perspective, as formulated in Equation (12):

(12) $$Y_{FCA}=IF\left(F(X_{Global})⊗Conv_{1}(GAP(X_{Global}))\right)$$

where F and IF, respectively, denote the Fast Fourier Transform (FFT) and its inverse transform. The output of the FCA module is denoted by $Y_{FCA}$, and global average pooling by GAP. Element-wise multiplication is indicated by ⊗. This process enhances inter-channel interactions and explores feature dependencies in the frequency domain. The resulting features are subsequently fed into a Spatial–Channel Attention (SCA) module, where convolutional operations are used to compress and redistribute the feature responses, as formulated in Equation (13):

(13) $$Y_{SCA}=Y_{FCA}⊗Conv_{1}(GAP(Y_{FCA}))$$

where $Y_{SCA}$ is the output of the SCA module, further highlighting key regions associated with small-face targets while suppressing irrelevant activations. Finally, the output features are processed by a Frequency-guided Spatial Attention (FSA) module. This module first integrates and transforms the input features through parallel convolutions, followed by an FFT-based mapping to the frequency domain, where global modulation is applied to different frequency components. This operation selectively enhances high-frequency information that is discriminative for small targets while attenuating background noise. The enhanced features are then restored to the spatial domain via IFFT and fused through a residual branch with adaptive weighting, injecting frequency-domain enhancement while preserving the stability of the original representations, as described in Equation (14), where $Y_{FSA}$ represents the output produced by the FSA module. Overall, the proposed module achieves effective coupling between frequency information and spatial structure, enabling the model to focus more reliably on critical details and thereby improving feature representation capability in complex scenarios.

(14) $$Y_{FSA}=IF\left(F\left(Conv_{1}(Y_{SCA})\right)⊗Conv_{1}(Y_{SCA})\right)$$

The scale complementarity of the three branches ensures that small-target cues are captured and aligned across different receptive fields, while the dual-domain attention mechanism performs denoising and feature enhancement prior to fusion. This design substantially improves the completeness and robustness of small-face feature representations in complex scenarios. Finally, the resulting features are aggregated with the outputs of the local signal modulation branch and the residual branch through element-wise addition, yielding the fused and enhanced representations for small-face targets.

## 4. Experiment and Results

### 4.1. Datasets

SCUT-HEAD: To evaluate the effectiveness of the proposed algorithm for face detection, we conduct training, validation, and testing on the publicly available SCUT-HEAD [36] benchmark dataset developed by South China University of Technology. The dataset consists of two subsets—namely Part A and Part B—and contains a total of 4405 images annotated with 111,251 bounding boxes. Part A includes 2000 images extracted from classroom surveillance videos, while Part B comprises 2405 images collected from complex classroom scenes on the Internet, covering a wide range of viewing angles and camera configurations. Notably, the SCUT-HEAD dataset provides a diverse collection of face instances with varying scales, poses, and levels of occlusion, which aligns well with the requirements of small-face detection addressed in this study. Experiments are conducted on the entire dataset, which is split into training, validation, and testing sets at ratio of 70%, 10%, and 20%, respectively. This split is used to evaluate the proposed model in comparison with other relevant methods.

WIDER FACE: WIDER FACE [37] is a widely adopted benchmark dataset in the field of face detection, encompassing 61 event categories, 32,203 images, and 393,703 annotated face instances. The dataset covers a broad range of face scales, including small, medium, and large faces. For each event category, the data are randomly split into 40% for training, 10% for validation, and 50% for testing. Based on factors such as face scale, occlusion level, and pose complexity, the WIDER FACE dataset further divides samples into three difficulty subsets: Easy, Medium, and Hard. Among them, the Hard subset is the most challenging and serves as an ideal benchmark for evaluating detection accuracy, particularly for small-face detection performance.

### 4.2. Experimental Environment

The proposed model is implemented using PyTorch 2.0.0 with CUDA 11.8. All experiments are conducted on a workstation running Ubuntu 20.04, equipped with an NVIDIA GeForce RTX 4090 GPU (24 GB of memory). The AdamW optimizer is employed in conjunction with a cosine annealing learning rate scheduler. The key experimental settings are summarized as follows: All input images are resized to 640 × 640. During training, the batch size is set to 8, and the model is trained for 200 epochs. The AdamW optimizer is configured with an initial learning rate of 1 × 10^−4^, a momentum value of 0.9, and a weight decay of 1 × 10^−4^. An IoU threshold of 0.7 is adopted, and the maximum number of detections is limited to 300. The random seed is fixed to 0, deterministic training is enabled, and four data loader workers are used.

### 4.3. Experimental Evaluation Metrics

The evaluation metrics [38] used in this study include precision (P), recall (R), average precision (AP), number of model parameters (Params), and number of floating-point operations (GFLOPs). At the same time, to better evaluate the ability of the model to detect small objects, we introduce the accuracy of small object detection (AP-s) index to measure the detection accuracy of small objects under the COCO index.

Precision (P) represents the proportion of all samples predicted as positive by the model that were actually positive and is calculated as follows:

(15) $$P=\frac{TP}{TP+FP}$$

Recall (R) represents the proportion of actual positive cases among all samples predicted as positive by the model and is calculated as follows:

(16) $$R=\frac{TP}{TP+FN}$$

Average Precision (AP) is a composite measure of the PR curve. mAP50 represents the average detection accuracy for all IoU threshold classes at 0.5 and is calculated as follows:

(17) $$AP=\int_{0}^{1}\;p(x)dx$$

(18) $$mAP=\frac{1}{k}\sum_{1}^{k}\;AP_{i}$$

Number of Model parameters (Params): This is often used to evaluate model complexity. A larger number of parameters may lead to a more symbolically expressive model and a better fit to the training data, but at the same time, it increases the risk of overfitting.

Number of Floating Point Operations (GFLOPs): This is a measure of the total number of floating-point operations performed by the model during inference or training. It is one of the metrics used to calculate the computational complexity of a model, which is used to evaluate the computational resource requirements and efficiency of the model.

Accuracy of small object detection (AP-s): This is used to measure the average detection accuracy of the model for small objects, which is the average detection accuracy when performing detection in steps of 0.05 for all IoU thresholds between 0.5 and 0.95.

### 4.4. Comparative Experimental Results and Analysis

#### 4.4.1. Comparative Experiments on Backbone Networks

As the core component for feature extraction, the backbone network plays a decisive role in the overall performance of small-face detection models. To evaluate the effectiveness of ISD-Net, comparative experiments are conducted using several advanced backbone architectures, with detailed configurations summarized in Table 1. The proposed ISD-Net consistently outperforms other state-of-the-art backbone modules in terms of both mAP50 and AP-s, while maintaining advantages in parameter efficiency and computational cost. Compared with the baseline model, the proposed method achieves an improvement of 1.1 percentage points in mAP50 and 1.1 percentage points in AP-s, while reducing the number of model parameters by 29.6% and decreasing GFLOPs by 16.8%. These results demonstrate that the ISD-Net backbone effectively optimizes computational redundancy and reduces model complexity, thereby providing a robust and efficient computational framework for subsequent network components.

#### 4.4.2. Performance Analysis of the MHMSA Module

**Figure 9:**
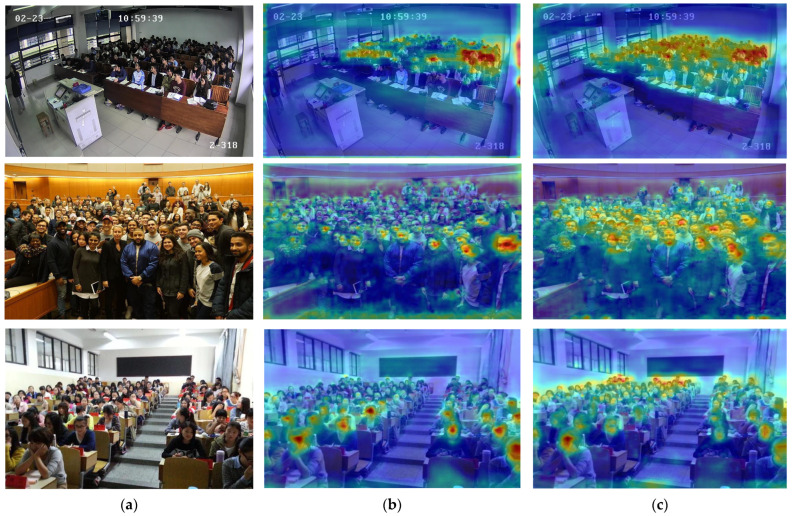
Heatmap comparisons with and without MHMSA. (**a**) GT; (**b**) RT-DETE-R18 (**c**) MHMSA.

Heatmaps are commonly used as a visualization tool in object detection to intuitively illustrate performance differences between models. Figure 9 presents a comparative visualization of the proposed MHMSA module and the baseline model. In the heatmaps, red and yellow regions indicate high attention weights, whereas blue and green regions correspond to low-weight responses. Variations in the attention distribution directly reflect the model’s ability to focus on target regions. Compared with the baseline model, MHMSA exhibits stronger attention responses for faces at relatively longer distances, with denser high-weight (red) coverage over neighboring face regions, indicating a significant improvement in spatial focusing accuracy. In contrast, the baseline model is more susceptible to complex background interference, often concentrating attention on larger faces located in the lower part of the image. By optimizing the attention mechanism, MHMSA effectively mitigates this issue, achieving more precise localization in upper small-target regions and preventing small-face features from being overwhelmed by background noise. Experimental results demonstrate that, under challenging conditions such as complex backgrounds, multi-scale distributions, and occlusions, the MHMSA module simultaneously enhances attention localization accuracy and target discriminability.

#### 4.4.3. Comparative Experiments on Feature Fusion Networks

In small-target detection, a common practice is to extend the feature pyramid from three to four levels by introducing an additional P2 detection layer. Although this strategy can improve detection accuracy, it comes at a considerable cost: the number of parameters increases by 7.5%, while the computational burden rises sharply by 70.2%, resulting in a substantially heavier model. To address this issue, we design the SFE-FPN architecture, in which the P2 layer is incorporated and subsequently fused with input features from the P3, P4, and P5 levels. In addition, the proposed CSOP-Fusion module is introduced, leading to only a 2% increase in parameters and a 14% increase in computational cost, thereby effectively reducing model complexity, as reported in Table 2. Despite its lower parameter count and computational overhead, the SFE-FPN architecture achieves notable performance gains. Compared with the conventional four-level pyramid model, it improves mAP50 by 1.6% and AP-s by 1.7%.

#### 4.4.4. Comprehensive Analysis of SFE-DETR

To comprehensively evaluate the effectiveness of the proposed SFE-DETR model for small-face detection, experiments are conducted on the SCUT-HEAD dataset. The proposed method is compared with several mainstream detection algorithms, including Faster R-CNN, SSD, YOLO series, and DETR-based methods. Six evaluation metrics are adopted: Precision (P), Recall (R), mAP50, AP-s, Params, and GFLOPs, which together provide a comprehensive assessment of detection performance. As reported in Table 3, the detection performance of Faster R-CNN, SSD, the YOLO series, and RT-DETR-R18 is significantly inferior to that of the proposed approach. Among YOLO-series models of comparable scale, although their overall performance appears competitive, the latest YOLOv11-m model achieves an mAP50 that is 1.1% lower than that of the proposed method, while the small-target detection metric AP-s is 1.7% lower. Although RT-DETR-R50 and Deformable DETR achieve competitive detection accuracy, they require substantially higher model complexity. Specifically, the mAP50 scores of RT-DETR-R50 and Deformable DETR are 0.6% and 1.1% lower, respectively, than that of the proposed method, while their AP-s values are 1.1% and 0.8% lower, respectively. In contrast, both models incur more than twice the number of parameters, and their computational costs are approximately two to three times higher. Compared with the baseline RT-DETR-R18, the proposed SFE-DETR achieves superior efficiency in terms of both parameter count and computational complexity, while improving mAP50 by 3.6% and AP-s by 3.1%, respectively. These results convincingly demonstrate the superior performance of the proposed method for small-face detection.

To comprehensively evaluate the generalization performance of the proposed SFE-DETR model for small-face detection, additional experiments are conducted on the WIDER FACE dataset. The proposed method is compared with mainstream face detection models based on ResNet and YOLO architectures, as listed in Table 4, using mAP50, Params, and GFLOPs as evaluation metrics. As shown in Table 4, although the YOLOv8Face model achieves slightly higher detection accuracy on the Easy and Medium subsets of the WIDER FACE validation set, the proposed method demonstrates clear advantages in challenging scenarios dominated by small faces. Specifically, on the Hard subset, the proposed model attains a detection accuracy of 86.31%, outperforming YOLOv8Face by 2.21 percentage points and surpassing the second-best model, YOLOv5Face, by 1.03 percentage points. Further comparisons with Face R-CNN, DSFD, RetinaFace, and TinaFace indicate that the proposed method consistently achieves the highest detection accuracy across the Easy, Medium, and Hard subsets. In particular, on the Hard subset, the proposed model improves performance by 14.92%, 22.14%, and 4.88%, respectively, compared to these methods. Even when compared with Transformer-based detectors, the proposed approach maintains strong performance on the Hard subset, demonstrating its significant advantages in both detection accuracy and computational efficiency.

In summary, the proposed algorithm achieves the best overall performance in comparative experiments on both datasets. Compared with models of similar scale, the proposed approach not only significantly reduces the number of parameters and computational cost but also improves both overall detection accuracy and small-target detection performance. These advantages make the proposed method particularly well suited for small-face detection in complex real-world scenarios.

### 4.5. Ablation Experiments

To evaluate the contribution of each module to the overall model performance, comprehensive ablation experiments are conducted. To ensure the reliability of the experimental results on the SCUT-HEAD dataset, five evaluation metrics are adopted: mAP50, AP-s, Params, GFLOPs, and FPS. The detailed results are summarized in Table 5. As shown in Table 5, Model 2 replaces the original backbone with the redesigned ISD-Net, resulting in a 0.9% improvement in mAP50, while reducing the number of parameters by 29.6% and the GFLOPs by 16.8%. Model 3 substitutes the conventional AIFI module with the proposed Multi-Head Multi-Scale Self-Attention (MHMSA) mechanism. By leveraging multi-scale feature extraction to capture richer representations, Model 3 achieves a 0.5% increase in mAP50 and a 1.4% improvement in AP-s. Compared with the traditional strategy of directly adding a P2 detection layer, Model 4 introduces the P2 feature map processed by SPDConv to generate small-target-aware features, which are then fused with P3 features and further integrated through the CSPO-Fusion module. This design enables effective learning of feature representations from global to local scales, leading to improvements of 1.0% in mAP50 and 1.9% in AP-s, thereby validating the effectiveness of the design introduced in Model 3. Model 5 incorporates the MHMSA mechanism into the original baseline (Model 1), yielding further gains in detection accuracy. Model 6, built upon Model 2, additionally integrates the proposed SFE-FPN, achieving further improvements in both mAP50 and AP-s compared with Model 2. Overall, when comparing the final configuration Model 7 (Ours) with the baseline model, mAP50 and AP-s are improved by 2.1% and 3.1%, respectively. Meanwhile, the proposed model reduces the number of parameters by 28.1% and decreases floating-point operations by 3%, demonstrating a favorable balance between detection accuracy and computational efficiency.

Table 6 presents the detection accuracy of the baseline model and the proposed approach on the WIDER FACE dataset, evaluated using mAP50, Params, and GFLOPs. As indicated by the experimental results, Model 2, which replaces the original backbone with the redesigned ISD-Net, achieves improved detection accuracy across the Easy, Medium, and Hard subsets, while reducing the number of parameters by 29.6% and decreasing the computational cost by 16.8%. The remaining improved models also demonstrate corresponding performance gains. Overall, after integrating the three proposed improvements, Model 4 (Ours) outperforms the baseline model by 2.2%, 1.5%, and 2.5% on the Easy, Medium, and Hard subsets, respectively. At the same time, the number of parameters is reduced by 28.1%, and the number of floating-point operations is decreased by 3%. These results indicate that the proposed model enhances small-face detection capability without compromising the detection accuracy for large faces.

Based on the ablation results obtained from both datasets, the proposed improvements consistently contribute to performance gains, demonstrating that the enhanced model is capable of effectively addressing small-face detection under complex conditions.

### 4.6. Visual Analysis

**Figure 10:**
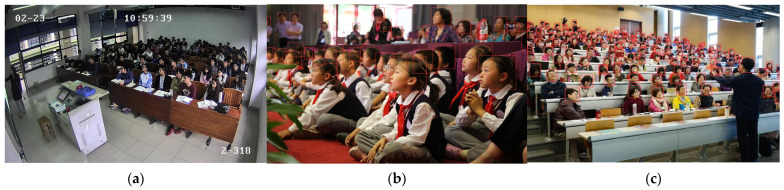
Example detection results of SFE-DETR, (**a**) high density, (**b**) shelter, (**c**) blur.

To validate the effectiveness of the proposed algorithm in real-world scenarios, representative images containing typical challenges such as dense distributions, occlusions, and blur are selected from the test sets of the SCUT-HEAD and WIDER FACE datasets. The face targets in these images are predominantly small in scale, and the corresponding detection results are illustrated in Figure 10. Figure 10a shows the detection results in dense scenarios. Despite the presence of a large number of overlapping face instances, the proposed algorithm successfully identifies the majority of faces with high accuracy. Figure 10b presents the results under partial occlusion conditions, where facial details are missing and visual cues are limited due to mutual occlusions. Even in such cases, the algorithm is able to reliably detect all face targets. Figure 10c demonstrates the detection performance in blurred scenarios, where images are affected by blur, occlusion, and illumination variations. The model not only accurately captures face targets but also successfully detects overlapping and blurred small-face instances. These qualitative results demonstrate that the proposed algorithm exhibits strong robustness and is capable of effectively handling diverse challenges encountered in real-world face detection scenarios.

**Figure 11:**
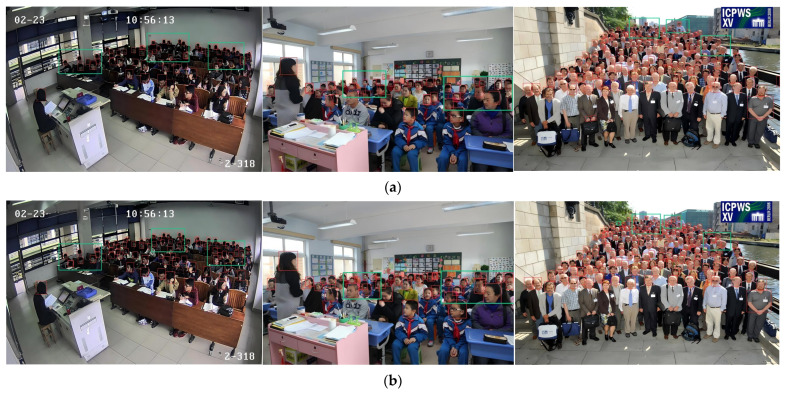
Comparison of the detection effects, (**a**) RT-DETR-R18 and (**b**) SFE-DETR.

To further verify the effectiveness of the proposed SFE-DETR algorithm, several representative images from dense and occluded scenarios are selected from the test sets of the SCUT-HEAD and WIDER FACE datasets and compared with the baseline method. As shown in Figure 11, due to the small target size and severe occlusions, the baseline algorithm fails to detect a portion of face instances, particularly near image boundaries and in densely populated regions. In contrast, the proposed SFE-DETR algorithm is able to accurately detect all targets even under heavy occlusion conditions. The detection results further indicate that, owing to the small scale and high density of the targets, the baseline method exhibits a high miss rate in the upper dense regions of the images. By comparison, the proposed approach not only successfully identifies the small targets missed by the baseline algorithm but also significantly reduces the overall miss-detection rate. Specifically, for small faces located at image boundaries and in densely crowded areas, the baseline method suffers from evident missed and false detections, whereas SFE-DETR effectively detects these challenging targets.

**Figure 12:**
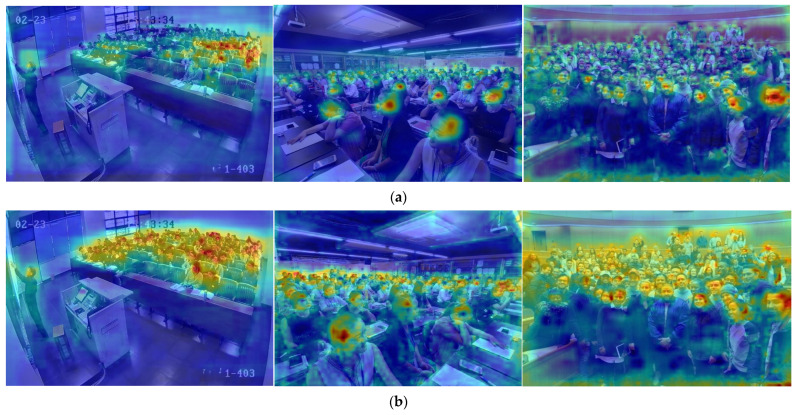
Comparison of heatmap visualization of RT-DETR-R18 (**a**) and SFE-DETR (**b**).

Heatmaps are a commonly used visualization technique in object detection, providing an intuitive representation of the spatial distribution of model responses over the input image. In the heatmaps, red and yellow regions indicate high attention weights, whereas blue and green regions correspond to low-weight responses. Differences in the weight distribution directly reflect the model’s ability to focus on target regions. Figure 12 presents a comparison of heatmaps generated by the RT-DETR-R18 model and the proposed method. In Figure 12a, the heatmap of RT-DETR-R18 shows that high-response regions are scattered around large face instances, failing to precisely capture the structural characteristics of the targets. In contrast, as illustrated in Figure 12b, the heatmap produced by the proposed model exhibits concentrated high-response regions over small-face targets in the background while simultaneously providing complete coverage of large foreground faces. More importantly, the proposed model demonstrates a markedly stronger focus on small-face instances. These results indicate that, compared with RT-DETR-R18, the proposed approach offers superior performance for small-face detection in complex scenes.

## 5. Conclusions

Aiming at the common challenges of small and medium-sized face target recognition in open and complex scenes, as well as the problem of insufficient accuracy in occluded target detection, this study proposes the SFE-DETR algorithm, which achieves better detection performance without significantly increasing computational cost. By constructing a new ISD-Net backbone network, the feature representation capability is effectively enhanced while reducing the number of model parameters. Second, the MHMSA mechanism is introduced to strengthen the ability to capture multi-scale target details and effectively suppress background interference in complex scenes. Finally, the SFE-FPN structure is designed to incorporate features from the P2 layer, where small target features are efficiently enhanced using large-kernel convolutions and dual-domain attention mechanisms. Experimental results show that the proposed model performs on par with or even surpasses existing detectors on the SCUT-HEAD and WIDER FACE datasets. These results fully demonstrate the superior advantages of our architecture, particularly in detecting small faces. However, there are still some limitations in this algorithm; future work could further explore collaborative optimization among modules to improve the model’s adaptability to extreme environments and dynamic scenes.

## Figures and Tables

**Table 1:** Results of comparative experiments on different backbone networks.

Models	P (%)	R (%)	mAP50 (%)	AP-s (%)	Param (M)	GFLOPs (G)
Base	90.2	86.3	92.6	39.0	19.9	57.0
DCNv2 [39]	91.6	88.1	93.2	39.6	20.2	53.7
DySnake [40]	91.9	88.9	93.4	39.8	29.9	65.1
IRMB [41]	90.7	86.6	92.9	39.5	16.7	53.4
DualConv [42]	90.8	87.4	93.1	39.7	16.2	52.5
Ours	91.5	88.3	93.5	40.1	14.0	47.4

**Table 2:** Results of the comparative experiments on different feature fusion network structures.

Models	P (%)	R (%)	mAP50 (%)	AP-s (%)	Param (M)	GFLOPs (G)
Base	90.2	86.3	92.6	39.0	19.9	57.0
Base + P2	89.6	87.7	93.4	40.1	21.4	97.0
SFE-FPN	91.9	88.9	93.6	40.9	20.3	65.1

**Table 3:** Comparison of SFE-DETR with other models on the SCUT-HEAD dataset.

Models	P (%)	R (%)	Map50 (%)	AP-s (%)	Param (M)	GFLOPs (G)
Faster R-CNN [4]	86.7	80.2	87.9	37.7	17.1	27.8
SSD [6]	84.3	68.7	86.3	38.1	13.3	22.8
YOLOv5s	91.1	85.1	92.5	39.0	9.1	24.0
YOLOv5m	91.8	87.9	93.4	39.8	25.1	64.2
YOLOv8s	91.0	85.8	92.4	38.9	11.2	28.6
YOLOv8m	92.2	88.3	93.6	40.5	25.9	78.9
YOLOv10s [43]	91.2	86.0	92.5	39.1	7.2	21.6
YOLOv10m [43]	92.3	88.9	93.7	40.2	15.4	59.1
YOLOv11s [44]	91.3	86.1	92.7	38.8	9.4	21.5
YOLOv11m [44]	92.5	88.6	93.6	40.4	20.0	67.6
RT-DETR-R18 [11]	90.2	86.3	92.6	39.0	19.9	57.0
RT-DETR-R50 [11]	92.7	90.9	94.1	41.0	42.0	136.0
Deformable-DETR [28]	92.1	90.3	93.6	41.3	40.0	173.0
Ours	92.6	90.7	94.7	42.1	14.3	55.2

**Table 4:** Comparison of SFE-DETR with other models on the WIDER FACE dataset.

Models	Backbone	Easy (%)	Medium (%)	Hard (%)	Params (M)	GFLOPs (G)
Face R-CNN [45]	ResNet152	93.7	92.1	83.1	-	-
DSFD [38]	ResNet152	94.2	91.4	71.3	120.0	259.5
RetinaFace [46]	ResNet50	94.9	91.9	64.1	29.5	37.5
TinaFace [47]	ResNet50	95.6	94.2	81.4	37.9	172.9
YOLO5Face [23]	YOLOv5-CSPNet	95.3	93.7	85.2	21.1	18.1
YOLO8Face [23]	YOLOv8-CSPNet	96.6	95.0	84.7	-	-
ADYOLOv5-Face [48]	YOLOv5-CSPNet	94.8	93.7	84.3	10.1	22.8
RT-DETR-R18 [11]	ResNet18	94.1	93.4	83.7	19.9	57.0
Deformable-DETR [28]	ResNet50	95.4	94.3	84.6	40.0	173.0
Efficient DETR [27]	ResNet50	95.9	94.2	85.1	32.0	159.0
Ours	ISD-Net	96.3	94.9	86.3	14.3	55.2

**Table 5:** Ablation experiments on the SCUT-HEAD dataset.

Methods	IRSD	MHMSA	SOEP-FPN	mAP50(%)	AP-s (%)	Param (M)	GFLOPs (G)	FPS
1. Base				92.6	39.0	19.9	57.0	64.2
2	✓			93.5	40.1	14.0	47.4	52.5
3		✓		93.1	40.4	20.0	57.5	59.2
4			✓	93.6	40.9	20.3	65.1	54.2
5	✓	✓		93.8	41.0	14.0	47.5	56.3
6	✓		✓	94.1	41.3	14.4	55.0	55.4
7. Ours	✓	✓	✓	94.7	42.1	14.3	55.2	61.7

**Table 6:** Ablation experiments on the WIDER FACE dataset.

Methods	IRSD	MHMSA	SOEP-FPN	Easy (%)	Medium (%)	Hard (%)	Params (M)	GFLOPs (G)
1. base				94.1	93.4	83.7	19.9	57.0
2	✓			94.9	93.7	84.1	14.0	47.4
3	✓	✓		95.5	94.0	85.2	14.0	47.5
4. Ours	✓	✓	✓	96.3	94.9	86.3	14.3	55.2

## Data Availability

The data and the conclusions of this study are available from the corresponding author upon reasonable request.

## Abbreviations

The following abbreviations are used in this manuscript:

P2, P3, P4, P5	Detection heads of different scales
MHMSA	Multi-Head Multi-Scale Self-Attention
MSA	Multihead Self-Attention
AIFI	Attention-based Intra-scale Feature Interaction
IoU	Intersection of Union
